# The Effects of Thiacloprid on Essential Components of Navigation and Pollination in Bumble Bees: A Laboratory Approach

**DOI:** 10.3390/insects17060651

**Published:** 2026-06-20

**Authors:** Inga Fuchs, Randolf Menzel

**Affiliations:** Institute of Biology/Neurobiology, Freie Universität Berlin, Königin Luisestr. 1-3, 14195 Berlin, Germany; inga.fuchs@fn.de

**Keywords:** neonicotinoid thiacloprid, exploratory learning, classical conditioning, landscape memory, match/mismatch tests

## Abstract

We developed a laboratory-based setup to perform behavioral tests of the effect of the neonicotinoid insecticide Thiacloprid in the CALYPSO^®^ formulation on bumblebees. This setup simulates essential components of navigation and pollination under natural conditions. The behavioral components are exploration, exploratory learning, learning of a rewarded local cue in the context of a specific panorama, and retrieving the memory for this association. The walking bumblebees navigated under their own motivation between a fully functional colony and a training/test arena. They explored the arena and learned the association of a rewarded local cue in the context of a panorama. The rule of association was that the local cue was bound to a particular part of the panorama irrespective of where it appeared in its spatial relation to the entrance gate through which the animal came from the colony. Extinction tests were performed for two conditions, match and mismatch. The match condition resembled the training condition. In the mismatch condition the local cue appeared in a different part of the panorama. Solving this task requires the learning and remembering of a rule under variable conditions, mimicking the cognitive requirements faced by bumblebees under natural conditions. The control animals solved this task, whereas animals treated with Thiacloprid 400 ng CALYPSO^®^ diluted in 4 µL per animal were significantly compromised, as shown by several parameters of the walking trajectories under the match and mismatch conditions. No dose–response functions were established, but a volume of 800 ng CALYPSO^®^ diluted in 8 µL per animal did not show any significant differences from a volume of 4 µL CALYPSO^®^. The setup and the experimental paradigm are suitable for routine quantitative tests on the effects of insecticides on the cognitive faculties of insects during navigation and pollination.

## Simple Summary

The study of the effects of insecticides require laboratory tests that can be applied as routine methods. We developed a laboratory-based setup to perform behavioral tests of the effect on bumblebees of the neonicotinoid insecticide Thiacloprid in the CALYPSO^®^ formulation. Walking bumblebees navigated under their own motivation between a fully functional colony and a training/test arena. They explored the arena and learned the association of a rewarded local cue in the context of a panorama. Solving this task requires the learning and remembering of a rule under variable conditions, mimicking the cognitive requirements faced by bumblebees under natural conditions. The control animals solved this task, whereas the animals treated with Thiacloprid in 4 µL (400 ng) CALYPSO^®^ were significantly compromised, as shown by several parameters of the walking trajectories under the match and mismatch conditions. No dose–response functions were tested, but a volume of 8 µL (800 ng) CALYPSO^®^ did not show any significant differences from a volume of 4 µL CALYPSO^®^.

## 1. Introduction

Neonicotinoids were invented in the 1980s as effective agents against insect pests. They are extensively used and abundant worldwide [1,2]. Seed coating and spraying with neonicotinoids are highly effective against sucking pest insects such as whiteflies, thrips and aphids [3]. The extensive use of neonicotinoids poses a threat to pollinating insects [1,4,5,6,7] and has led to a ban on three highly toxic neonicotinoids in Europe [8]. The less toxic neonicotinoid Thiacloprid [9] has also been banned, but its application in agriculture continues to compromise important natural behaviors of pollinators (such as foraging activity, homing behavior, learning, memory formation and retrieval in honeybees, [10,11,12,13,14]; for a recent review see [15]). In bumblebees, Ellis et al. [16] found a striking effect (reduction by 46%) on the efficiency of affected colonies in producing reproductives compared to control colonies. Similar effects were found for honeybees [17,18]. Affected colonies were also much lighter in weight, suggesting disturbed foraging activity. These and other effects call for further studies of the sublethal effects of Thiacloprid in the CALYPSO^®^ formulation since special permits are multiply issued, and Thiacloprid is still used in countries outside the European Union.

Neonicotinoids bind the postsynaptic nicotinic acetylcholine receptor (nAChR). Their function is based on the permanent docking of the active agent on the nAChR located in the central nervous system of insects, disturbing the normal transmission of nerve impulses by continuous stimulation, causing severe muscular impairment [19,20]. Their agonistic action induces continuous excitation of the postsynaptic membrane, producing discharges leading to cell energy exhaustion, paralysis and death [21]. nACh receptors are abundant in the brain centers of insects, in particular in mushroom bodies known to be involved in higher-order sensory integration, neural plasticity, learning, memory, motivation and goal directedness [22]. Laboratory tests are required to reveal the detrimental effects of formulations based on Thiacloprid, such as CALYPSO^®^. The behavioral test procedures reported here allow us to quantify this high-order neural processing in a laboratory setting that simulates the basic navigational performance of freely walking bumblebees. Our study aims to characterize the detrimental effects of Thiacloprid at the sensory, motor, motivational, learning and memory-retrieval level by applying laboratory test procedures that can be included in routine tests. We find sublethal effects predominantly at the level of insect cognition that is essential for navigation and pollination under natural conditions.

## 2. Material and Methods

### 2.1. Animals and Setup

**Figure 1:**
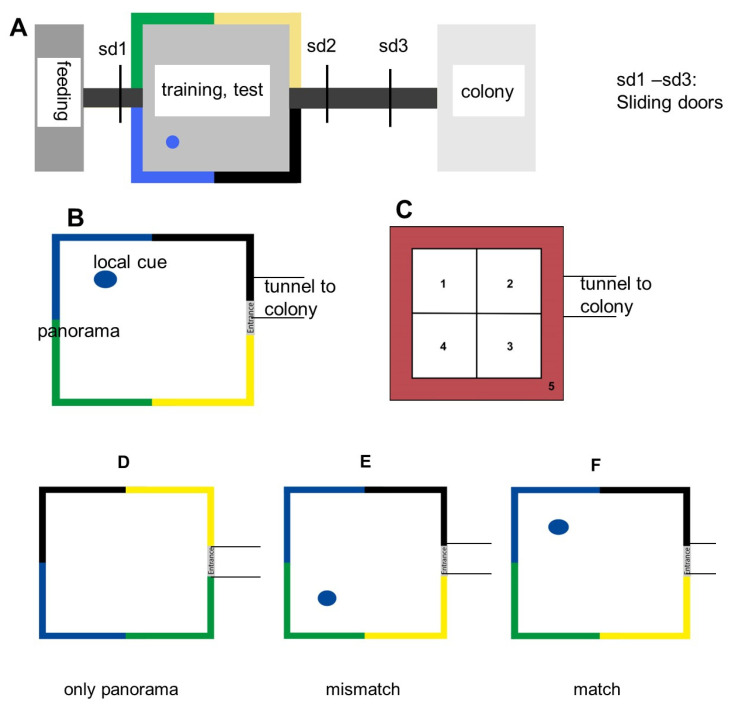
Behavioral setup. (**A**): Colony box, training/test arena (from **right** to **left**). The boxes are connected by tubes. The feeding box is accessible during pretraining. The training/test arena in the middle shows the training situation (match between local cue and panorama). The blue local cue contains sucrose solution during training and lacks sucrose solution during the test (extinction test). (**B**): The arrangement of panorama and local cue relative to the tube from the colony changes between training sessions and test (compare (**A**,**B**)). (**C**): Definition of the five areas that occupy the same area size. (**D**–**F**): Arrangements during the three different test conditions.

The setup consisted of three parts, the box with the colony, the training and test arena, and the feeding (pretraining) arena (Figure 1). The bumblebee colonies were procured from Katz Biotech AG (Baruth, Germany). They were approximately 10 weeks old and consisted of 60–90 worker bees plus the queen. After arriving in the lab, the box was placed on a table for at least 20 min to establish a state of reduced stress. The queen was taken out to clip her wings with nail scissors and mark her. Pollen dough (powdered pollen mixed with water, pollen ordered at Heinrich Holtermann KG, Brockel, Germany) was placed around the combs, and, once prepared, the queen was put on top of the combs. Then worker bees were taken out one by one, and their wings were also clipped. By cutting both wing pairs, it was ensured that they did not try to fly. The bees became used to this non-flying state quite quickly and showed no impaired behavior, corroborating the observations by [23]. Once prepared, these workers were also placed on the combs. Sugar solution (1/1 sugar/water) was positioned in a feeder in the feeding arena. The colonies were kept under a 12 h light/dark cycle. The animals were allowed to explore their surroundings for 20 h before individual marking started. The wings of newly hatched bees were cut every morning, and the death and birth of animals were recorded. For the weekends, the feeder was replenished and placed in the feeding arena, and new pollen dough balls were put directly into the colony. During the training sessions, the feeding arena was closed to ensure that the bumblebees were hungry and motivated to forage, following the experience of [24,25]. Throughout the week, only pollen was offered, to keep the queen busy producing eggs and to ensure normal larval development. The colony was dismissed when the foragers exhibited aggressive behavior and ceased foraging completely. If (under any circumstance) the queen died, the colony was also dismissed because normal foraging behavior would disappear too.

The tags for individual marking were printed on photo paper to ensure stability and were subsequently punched out using a hole puncher. They consisted of five distinct colors with alphanumeric characters (33 of each color) at a size of 5 mm. Foragers were selected from the feeding site and were placed in a marking tube (Imgut^®^ Königinnen Zeichenrohr ordered at Heinrich Holtermann KG, Brockel, Germany). Fixed there, the thoracic hairs were shaved, and the tags were affixed onto the thorax with a minimal amount of super glue gel (UHU^®^ Sekundenkleber Supergel, UHU GmbH & Co KG, Bühl/Baden, Germany). The animals were put back into the arena after being kept in tubes for five minutes to allow the glue to dry adequately. This procedure was repeated every Monday morning to create enough marked foragers for subsequent training sessions until the first appearance of drones. At this stage, the marking was discontinued to ensure that only foragers were marked.

### 2.2. Training and Test

Training started always at the beginning of the week. The entrance to the arena was closed with two doors (Figure 1, sliding doors sd2 and 3). The two-barrier system ensured that only one bee at a time entered the arena. Two individually marked bees were selected for training. One as a control and one for treatment. These pairs of individuals came from different colonies. Sugar solution (1.5/1 sugar/water ratio) was used for the training process. Two pieces of filter paper soaked with farnesol were put in the arena to enhance foraging behavior [26,27]. Care was taken to ensure that no sugar solution was in the combs, so the bees were hungry and motivated to forage. Video recordings were performed using a Raspberry Pi 4 Camera Module v2, Raspberry Pi Ltd., Cambridge, UK). The panorama consisted of cardboard walls with colored vertical stripes with colors as marked in Figure 1. The colors (in human terms) were yellow, green, black, and blue. A painted game piece in royal blue served as the local cue that also contained the sucrose solution as a reward. During training the local cue was always placed in front of the same panorama color (blue). This arrangement is referred to as a “matching situation” or “match”. After training, the cat sand on the arena floor was freshly mixed, so no odor cues were present [28,29]. When the first bumblebee entered the arena the video recording was begun, and the test bee started to explore the arena ad libitum. When she returned to the entrance gate, doors sd2 and sd3 were opened, and she could join the colony. The cup (local cue with reward) and the panorama were rotated relative to the entrance gate, and the cat sand was thoroughly mixed to distribute any odor marks possibly left by the previous forager. The rotation ensured that the bee did not learn a particular arrangement of the local cue and the panorama in relation to the gate but the association between the blue local cue and the blue stripes of the panorama. The next bumblebee (the same bee as before or a new marked bee) could then enter the arena. A full protocol of the training sessions was established. Such training sessions usually ran for three hours on 3–4 consecutive days. Tests without the sucrose reward (extinction tests) were conducted with trained bees after each training session to examine the progress of the training. At the end of the week, three different test procedures were performed as explained below. Usually 2–4 bees were trained and tested per week. Different behaviors were quantified separately for the five areas (Figure 1D,E).

The test situations were as follows: panorama only, match (same conditions as during training), mismatch (the panorama and the local cue were located at different positions). Figure 1F shows the match situation. While testing, no sugar solution was provided (extinction test). All runs were video recorded, tracked and analyzed. The bees were allowed to enter the arena whenever they were motivated unless the arena was occupied by another bee. Importantly, the animals were not touched or moved by the experimenter at any time.

### 2.3. Video Analyses and Statistics

The videos of the behavioral tasks were recorded using software obtained from GitHub (https://github.com/Billwilliams1952/PiCameraApp, accessed on 15 June 2026). These videos were converted into the AVI (Audio Video Interleave) format. All videos were then tracked with the Biotracker of the Landgraf research group (FU Berlin, Robotics group). Further information about this tracker can be found here: https://github.com/BioroboticsLab/biotracker_core/wiki (accessed on 15 June 2026). The tracks were stored as CSV (comma-separated values) files and used for further analysis.

The analyses were carried out in RStudio (Version 2025.12.1, Posit Software PBC, Boston, MA, USA) using the R programming language. The corresponding statistical analyses were performed using GraphPad Prism (GraphPad Software, Inc., Boston, MA, USA, Version 11.0.0). All the groups were first checked for normality with a Kolmogorov–Smirnov test. The selected behavior parameters and the appropriate statistical tests are given in the figure legends. Heat maps of walking trajectories were visually inspected.

### 2.4. Treatment with CALYPSO^®^

*Two* groups of bees (the control and the CALYPSO^®^ group) were run in parallel. The animals from these two groups were handled individually by moving them into and out of separate handling and feeding tubes. Animals of the control group received 4 µL of sugar solution (1/1 sugar/water ratio, final sugar concentration by weight was 1/1 sugar/water ratio) per animal and were then kept for 1.5 h under red light. The treated animals were fed with 400 ng CALYPSO^®^ diluted in 4 µL per animal in sugar solution and handled in the same way. Preparatory experiments were performed with double the volume of Calypso as used throughout the experiments. No significant differences in the behavior of the bumble bees were found. The volume of 8 µL CALYPSO^®^ solution, however, was not always fully taken up. Therefore, we performed all experiments with 4 µL sugar solution or CALYPSO^®^ sugar solution. The handling tubes were video recorded for 1 h. This procedure allowed us to ensure that the animals digested the food and were not able to distribute it into the colony afterwards. It was also possible to detect any misbehavior (e.g., cramps or other unnatural behavior). The animals were then put into the colony again, and the testing followed the protocol described above. Usually, the animals appeared at the entrance gate within 30 min and were then allowed to enter the training/test arena.

### 2.5. Residue Analysis

Ten animals per time and dose group were carefully selected from a functional colony. These animals were then each fed separately with 400 ng or 800 ng CALYPSO^®^ diluted in 4 µL or 8 µL sugar/CALYPSO^®^ solution per animal (final sugar concentration 1/1 sugar/water ratio). Following ingestion, the animals were kept under red light for various time periods (1.5 h, 3 h, 4.5 h). After the time had elapsed, the animals were promptly frozen at −80 °C. Once completely frozen, three body parts (head, thorax, abdomen) were separated, packed, appropriately marked, and dispatched to the laboratory technician. The analyses were carried out by the Labor Friedle GmbH (Tegernheim, Germany, https://www.labor-friedle.de, accessed on 19 June 2026), a laboratory specialized in conducting such analyses. The Thiacloprid concentrations reached saturation after 1.5 h. Most Thiacloprid was detected in the abdomen (1000 µg/k). A total of 200 µg/kg were found in the head and 50 µg/kg in the thorax (Figure S1).

## 3. Results

### 3.1. Spontaneous Preference and Learning

**Figure 2:**
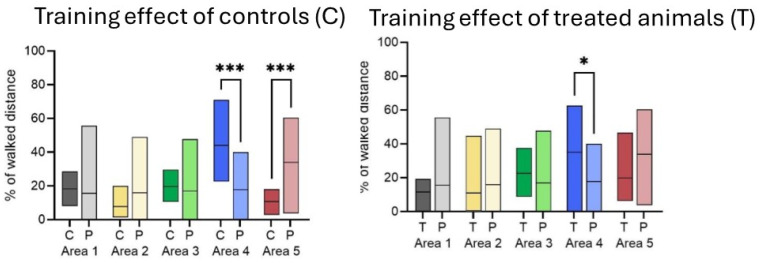
Training effect on controls (C) and on treated animals (T). The boxes show the mean walking distance in all five areas before training (P: spontaneous preference test) and after training (C: extinction test) for control animals and treated animals (T: treated, n = 9, and P: spontaneous preference test, n = 9). The dark lines in the bars give the mean. Asterisks mark significant differences (*t*-test with Welch correction, significance levels: * *p* < 0.05; *** *p* < 0.001). No significant differences if no significance ranges are given. The colors of the plots represent the colors of the panorama in that specific area. The blue bars show the situation of the matching local cue and panorama. Notice the higher significance level for the matching area (here area 4) in the control animals but lower level in treated animals.

First, we addressed the question of whether Thiacloprid treatment alters the spontaneous preferences, exploratory behavior, and exploratory learning of the bees (Figure 2). Exploration is essential to the animal learning about the rules that it must acquire when discovering a new feeding site, here the sucrose reward at the local cue in combination with the panorama. Differences in walking distance in the five areas captures well the spontaneous choice before exploratory learning and after exploratory learning. A highly significant difference was found between control and treated animals for area 5, the area that animals choose when they are in an escape mood. This result shows that Thiacloprid has no effect on sensory and motor performance, but has an effect on the tendency to escape. Initially, both the control and the treated animals choose area 5 equally strongly, but after exploration control animals choose it to a very low degree, whereas the treated animals do not change their behavior, indicating differences in the process of becoming familiar with the environment during exploratory learning. Learning leads to a highly significant increase in walking distance in the area of the rewarding local cue. A small effect of training is seen also in treated animals.

**Figure 3:**
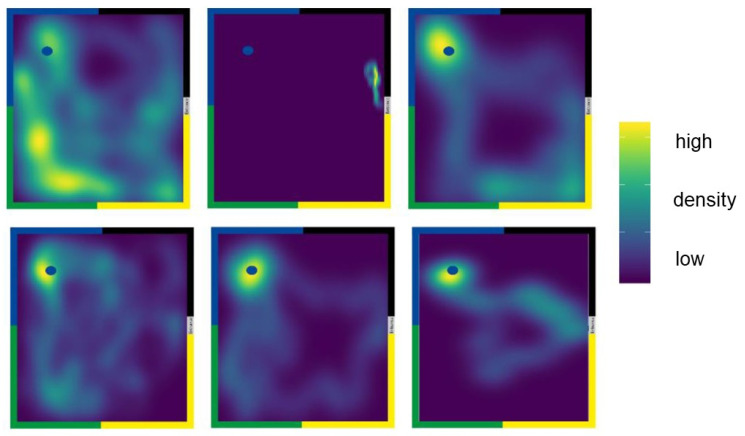
Heat maps of three representative examples of walking trajectories after training during extinction tests (no food available at the local cue). The upper row shows the treated animals, the lower row the control animals. The frame gives the panorama, and the blue the location of the local cue. The small gray bar in the middle of the right side marks the entrance gate to the area. No food is provided at the local cue during these extinction tests.

Figure 3 gives three exemplary heat maps of walking trajectories for each of the control and the treated animals during extinction tests. Control animals searched specifically in the area of the local cue and the panorama associated with the local cue, whereas the search trajectories of treated animals were much less focused on the correct area or were even not performed.

### 3.2. Match–Mismatch Tests

**Figure 4:**
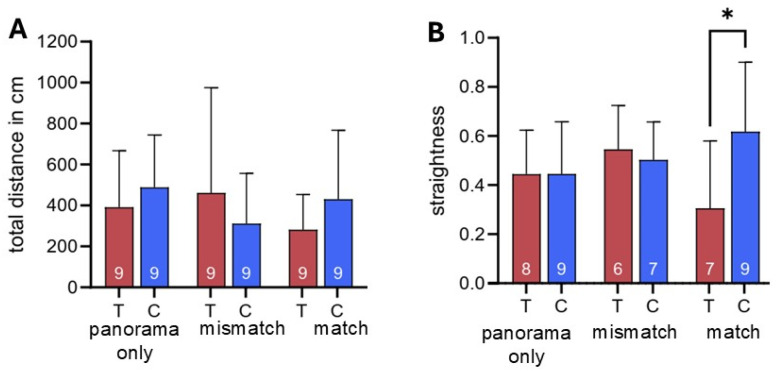
Total length and straightness of the walking trajectories. The plots represent the mean of the total length of the walking trajectory and the straightness of the trajectory, with the standard deviation for the tests of the two groups (treated T, red, and control C, blue). The number of animals is marked within the bars. Asterisks mark significance. Three test conditions are indicated: panorama only; mismatch (panorama and local cue located at different positions); match (panorama and local cue coupled to each other, as in training). (**A**): Total length of the walking trajectory in the different tests. (**B**): Straightness of the two groups in the different tests. Significant difference in match test (* *p* = 0.04; *t*-test with Welch correction).

In the next series of experiments, animals were specifically trained to the matching conditions of the local cue and panorama by changing the appearance of the matching situation in different spatial relations to the entrance gate. Two parameters were calculated from the walking tracks of the experimental and control animals, the total distance and the straightness of the walks (Figure 4). Three tests were run: panorama only, mismatch (panorama and local cue located at different positions) and match (panorama and local cue coupled to each other, as during training). No significant difference was found in the total length of the walking trajectory. The straightness of the trajectory towards a goal was calculated by the ratio of the length of the direct route to the local cue or the panorama divided by the length of the actual route taken. The optimal straightness is 1. A significantly lower value for straightness (*p* = 0.04; *t*-test with Welch correction) was found in the treated animals for the match situation, indicating impairment in walking towards the learned goal (local cue and panorama) after uptake of Thiacloprid.

### 3.3. Memory Retrieval

**Figure 5:**
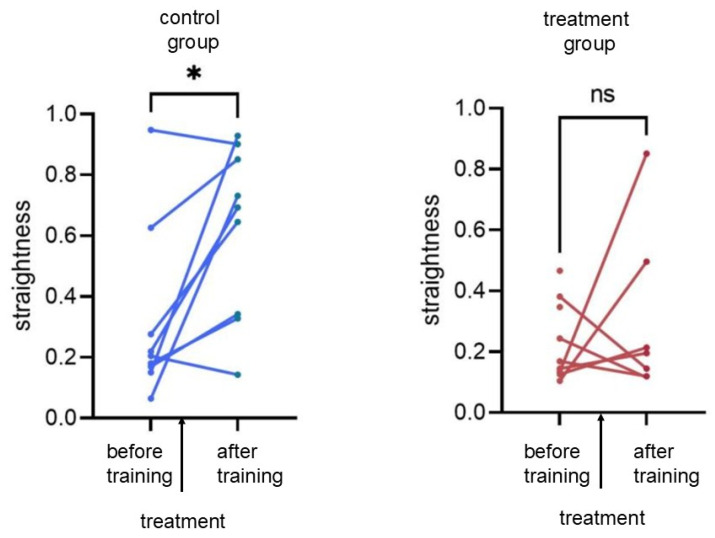
Comparison between the straightness of the walking trajectory during the first successful training run and the extinction. Statistical significance is shown by asterisk (* *p* = 0.02; Wilcoxon test). ns: no significant difference.

Next, the effect on memory retrieval was tested by comparing for each animal in the two groups the difference between the straightness of the trajectory before full training (the bees find the food in the first training trial) and after training (first extinction test with the local cue and panorama as during training) (Figure 5). The comparison thus uncovers the effect of CALYPSO^®^ on memory retrieval. The treated animals are impaired in retrieving their memory (*p* = 0.02, Wilcoxon test). These results corroborate those of Figure 4, emphasizing the sensitive parameter (straight walks toward the learned cue, the association of local cue and panorama irrespective of where this situation appears when entering the test arena through the gateway).

**Figure 6:**
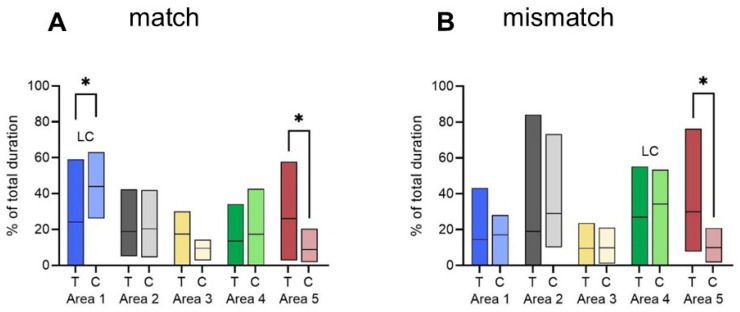
The probability of the total time spent in the five areas for the two groups (treated and control) in two test situations, match and mismatch. Number of animals in the treated group: n = 9; control animals: n = 9. The boxes give the whole range of data and the mean. Asterisks mark significant differences. The colors of the plots represent the colors of the panorama in that specific area. The position of the local cue is labeled (LC). The fifth (red) area depicts the border area. (**A**): Match. (**B**): Mismatch. Significant differences are marked (* *p* = 0.02, *t*-test with Welch correction).

A further comparison between the control and the treated animals focused on the time spent in each of the five areas (Figure 6). In both test conditions (match and mismatch), the treated animals stayed significantly longer in the border area (*p* = 0.02, *t*-test with Welch correction), indicating a higher tendency to escape from the arena, a finding that supports what is shown in Figure 2. In the match test situation, the control animals stayed significantly longer in the area that they had learned (the blue local cue is associated with the blue panorama). Thus, treated animals are shown to be compromised in recalling their memory (Figure 6A). We also tested the panorama only situation and found no significant differences between control and treated animals, indicating an effect specific to the local cue/panorama association.

**Figure 7:**
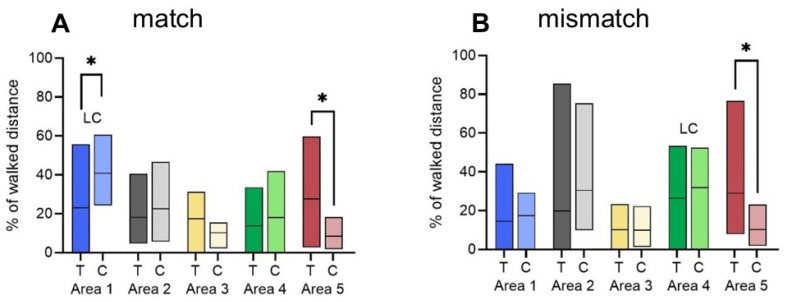
The percentage of the total length of the walking trajectory in the five areas for the two groups (treated: T and control: C) in two test situations, match and mismatch. The boxes show the percentage of the total length of the walking trajectory of the test run per area and test group (T: treated, n = 9; C: control, n = 9) from minimum to maximum with the mean as the hyphen. Asterisks mark significance. The colors of the plots represent the colors of the panorama in that specific area. The position of the local cue is labeled (LC). The fifth (red) area depicts the border area. (**A**): Test of match situation (with position of panorama and local cue coupled). Significance in area 1 (* *p* = 0.02, *t*-test with Welch correction). (**B**): Test of mismatch situation (with different positions of the panorama and local cue). Significance in area 5 (* *p* = 0.05, *t*-test with Welch correction) and area 5 (* *p* = 0.02, *t*-test with Welch correction).

Increased time spent in a particular area could result from slower walking or no walking at all. We therefore analyzed the walking time, excluding periods of not walking (Figure 7). Control animals performed as expected, walking for significantly longer in the area that contained the learned situation (here area 1). Treated animals walked less in area 1, the trained area, indicating that they remembered the trained conditions less well. Extensive training as performed here leads to significantly shorter walks in area 5 in control animals, but not in treated animals either in match or in mismatch situations. Interestingly, there is a tendency in both control and treated animals to walk longer in the area with the local cue (here area 4) in the mismatch situation (compare the data plotted in green bars in the match and mismatch situation). We also tested the panorama only situation and found no significant differences between control and treated animals. As pointed out above, this appears to emphasize the specificity of the learned conditions.

**Figure 8:**
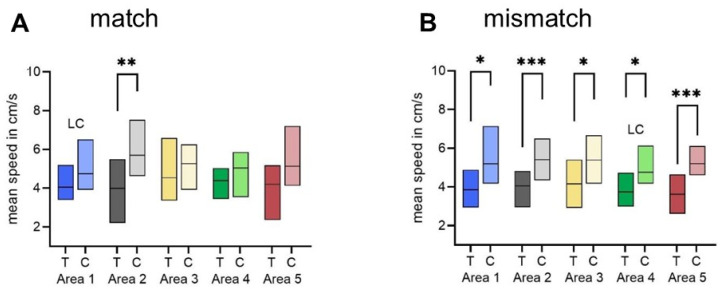
Mean walking speed in the five areas for the two groups (treated and control) in the two test situations, match and mismatch. The boxes show the mean speed of the test run per area and test group (T: Thiacloprid intake 400 ng, n = 9; C: control, n = 9) from minimum to maximum with the mean as the hyphen. Asterisks mark significance (t-test with Welch correction). The colors of the plots represent the colors of the panorama in that specific area. The position of the local cue is labeled (LC). The fifth (red) area depicts the border area. (**A**): Test of match situation. Significance in area 2 (** *p* = 0.004). (**B**): Test of mismatch situation. Significance in all areas (area 1: * *p* = 0.01; area 2: *** *p* < 0.001; area 3: * *p* = 0.02; area 4: * *p* = 0.02; area 5: *** *p* < 0.001).

Although it appears that Thiacloprid does not alter motor performance (see Figure 2) in our test conditions, we next wanted to evaluate the motivation to search for the reward during extinction tests (no reward) by analyzing the speed of walking in the two test conditions, match and mismatch. The mismatch test is of particular relevance here because the animals experience a situation in which the expected association between the local cue and panorama is not found. The walking speed values are significantly higher in control animals than in treated animals for all areas in the mismatch situation, indicating a higher motivation to resolve the unexpected situation. As expected, walking speed is higher in control animals than in treated animals when they experience the expected situation (match) (Figure 8).

## 4. Discussion

Neonicotinoids play an important role in the agricultural business. Their use has rapidly increased over the last few decades. They are very effective against their target pest insects, which include sucking insects such as aphids or whiteflies, but they are also taken up by non-target insects, including pollinating insects. The abundance of these pesticides is alarming as they can be found everywhere and not just in the treated plants. Other insects come into contact with these chemicals via consumption of contaminated pollen or nectar or even through direct acute contact. Chronic exposure and sublethal effects are already the subject of various studies on several neonicotinoids [7,30,31,32]. Although the four nicotinoids known to be the most toxic (Thiamethoxam, Imidacloprid, Acetamiprid and Clothianidin) are already banned in the EU, it is still important to evaluate their impact further because of the possibility of future re-admission. The detrimental effect of Thiacloprid is played down because of its higher LD_50_ value compared to other neonicotinoids [33], but the sublethal effects are known to interfere with navigation, learning and communication. Field relevant doses of Thiacloprid and/or its formulation CALYPSO^®^ are discussed at length in [12,16]. It is concluded that nonlethal doses 250–800 times lower than LD_50_ (LD_50_ for Thiacloprid: 17.32 μg/bee) as applied here impair learning and memory retrieval (see also Introduction and below).

The action of the neonicotinoid Thiacloprid is well understood both at the molecular and cellular level [19,34]. Here, the sublethal effect of Thiacloprid in the CALYPSO^®^ formulation was tested. In a study by [12], it was found that Thiacloprid in the CALYPSO^®^ formulation impaired natural navigation performance, the motivation to forage, and the performance of waggle dances in honeybees. We therefore selected CALYPSO^®^ as a pharmacological treatment affecting central nACh receptors to study the effect in a laboratory setting using our training and test procedure. The goals were twofold: (1) to establish a laboratory-based test procedure that simulates natural conditions during foraging and pollination, and (2) to explore the usefulness of the test procedure for routine tests of sublethal effects on insect cognition. The latter goal is particularly important in the context of the search for laboratory test conditions of sublethal effects. So far, the standard tests on the effect of insecticides have been LD_50_ tests, which seek to determine the 50% of dead animals after 24 h. The European Commission is actively searching for standard tests of sublethal effects that include the bumblebee as a test animal (personal commnication). In the tests we devised, the bumblebees were foraging under their own motivation. Additional time (1–2 h) elapsed until they entered the test arena, ensuring that the drug was completely absorbed in their body. Drug residue measurements in the head allowed us to make it likely that the drug reaches the brain during this period.

Our experimental setup required cutting the wings of all bees including the queen. Although the treated animals showed no differences with respect to the controls when their sensory and motor performance was analyzed (Figure 2), the lack of wing movement inside the colony certainly has an impact on social behavior (e.g., warming the brood). Learning about a new feeding place might even require normal motor pattern in flight and successful buzzing and wing vibration in the colony arousing other bees to take up foraging activity. In this sense, our experimental procedure reflects a compromise as compared to natural learning during foraging. Importantly, exploratory behavior involved in learning the location of a newly discovered feeding place was not found to be compromised. Furthermore, the results of treated animals were compared with untreated animals who were handled in the same way (wings cut, pre-exposure to feeding, manipulation for test condition). In addition, the match/mismatch test conditions were chosen to focus on a form of learning beyond classical conditioning (context-dependent learning). In the match situation, the straightness of the walks decreased in the treated animals whereas it increased in the control animals, indicating an effect on memory retrieval in the trained condition (Figure 5). This finding is in line with the results of [12] who found longer and less precise trajectories in homing flights. The learning of the task (the spatial association of the local cue with the panorama) was significantly reduced in treated animals (Figure 5 and Figure 6). In addition, the occurrence of longer walking trajectories in the border area (area 5) after CALYPSO^®^ treatment during initial exploration and no reduction in the walking trajectories in area 5 during learning indicated stronger escape behavior and reduced learning (Figure 6 and Figure 7). In the mismatch test, walking speed as a measure of the motivation to solve the discrepancy between the learned condition and the actually experienced condition is significantly lower in treated animals than in control animals in all test areas (Figure 8). Taken together, these results document the usefulness of the procedure in uncovering the detrimental effects of Thiacloprid on insect cognition, as previously found in experiments with freely flying honeybees [10,11,12,13,14].

## 5. Conclusions

The laboratory setting developed here allows for testing the action of insecticides on high-order cognitive functions in insects. We conclude that the laboratory setting is well suited to testing and quantifying the sublethal effects on navigation under laboratory conditions but needs to include several additional measures, e.g., dose effects, close comparison with doses to which bumble bees are exposed under agricultural conditions, tracing the drug into the brain and clearing phenomena. These detrimental effects can be attributed to the impaired retrieval of navigational memory, corroborating the results obtained under natural conditions. The setting also provides a tool to access memory retrieval phenomena by pharmacological methods. The results found here corroborate the importance of nicotinic transmission in the context of memory formation and retrieval.

## Data Availability

The original contributions presented in this study are included in the article and Supplementary Materials. Further inquiries can be directed to the corresponding author.

## Supplementary Materials

The following supporting information can be downloaded at https://www.mdpi.com/article/10.3390/insects17060651/s1. Figure S1. Residue analyses of Thiacloprid in three different body parts after the treatment.
