# Supplementary material for: The Effects of Thiacloprid on Essential Components of Navigation and Pollination in Bumble Bees: A Laboratory Approach
Source: Insects. 2026 Jun 20;17(6):651. doi: 10.3390/insects17060651 (PMC13300399; doi:10.3390/insects17060651)
Supplement: Supplementary file 1 [file insects-17-00651-s001.zip › insects-4317628-supplementary.pdf]

Supplement

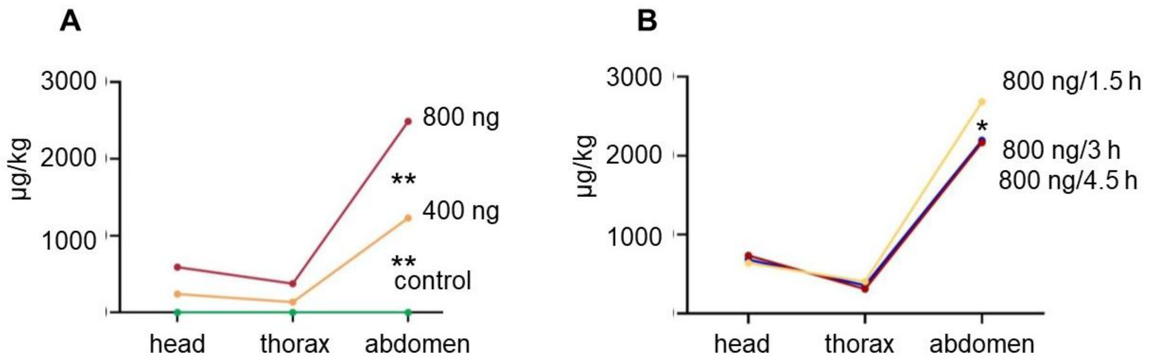

Figure S1. **Residue analyses of Thiacloprid in three different body parts after the treatment. A**: The concentration (µg/kg) of Thiacloprid in the 3 body parts (head, thorax, abdomen) of control (no intake, green line), 400 ng (yellow line) and 800 ng (red line) intake of Thiacloprid. Animals (n = 10 per group) were frozen after 1,5 h of ingestion under red light. **B:** The concentration (µg/kg) of Thiacloprid in the 3 body parts (head, thorax, abdomen) of 800 ng intake of Thiacloprid. Animals (n = 10 per group) were frozen after 1,5 h (yellow line), 3 h (blue line, fully overlapping with red line) and 4,5 h (red line) of ingestion under red light. The dose in the abdomen decreased over time between ingestion and measurement. The dose in the head and thorax show no clear tendency for the ingestion time. The dose dependence in the head is the relevant finding documenting that Thiacloprid reaches the head already after 1,5 hours, most likely predominantly the brain, judging from the volumes of tissue in the head. Significance levels: * <0.05, ** <0,005.

.
